# Low-Cost, Disposable Biosensor for Detection of the Brain-Derived Neurotrophic Factor Biomarker in Noninvasively Collected Saliva toward Diagnosis of Mental Disorders

**DOI:** 10.1021/acspolymersau.5c00038

**Published:** 2025-08-01

**Authors:** Nathalia O. Gomes, Marcelo L. Calegaro, Luiz Henrique C. Mattoso, Sergio A. S. Machado, Osvaldo N. Oliveira, Paulo A. Raymundo-Pereira

**Affiliations:** † Sao Carlos Institute of Chemistry, IQSC−USP, University of Sao Paulo, 13566-590 Sao Carlos, São Paulo, Brazil; ‡ Nanotechnology National Laboratory for Agribusiness (LNNA), Embrapa Instrumentation, 13561-206 São Carlos, São Paulo, Brazil; § Sao Carlos Institute of Physics, IFSC−USP, University of Sao Paulo, 13566-590 Sao Carlos, São Paulo, Brazil

**Keywords:** major depressive disorder (MDD), BDNF, diagnosis, disposable biosensors, human saliva, therapeutic drug monitoring

## Abstract

The importance of early detection of neurodegenerative disorder biomarkers has grown since these biomarkers are essential for timely diagnosis, treatment, healthcare, and wellness applications. We present a cost-effective and disposable electrochemical immunosensing strip for rapid, decentralized detection of brain-derived neurotrophic factor (BDNF)—one of the major neurotrophins (NTs) associated with neurological and psychiatric disorders—in human saliva. The salivary BDNF immunosensor strip is made on a screen-printed carbon electrode functionalized with carbon spherical shells (CSSs), polyethylenimine (PEI), and glutaraldehyde to enhance sensitivity. Through systematic optimization, the sensor achieved excellent analytical performance, with a wide dynamic detection range from 1.0 × 10^–20^ to 1.0 × 10^–10^ g mL^–1^, a rapid response time of under 3 min, and an ultralow detection limit of 1.0 × 10^–20^ g mL^–1^ for BDNF in human saliva. The BDNF immunosensor demonstrated high selectivity, reproducibility, robustness, stability, and long-term storage capability. At a cost of less than US$ 2.19 per unit, this disposable sensor also enabled rapid BDNF detection in saliva samples collected from healthy volunteers without interference from other salivary constituents. The environmental impact was assessed using the Analytical Eco-Scale (AES), the Analytical GREEnness Metric Approach (AGREE), and the Blue Applicability Grade Index (BAGI), which evaluates the practicality (“blueness”) of the device. These assessments confirmed the sustainability of the disposable BDNF immunosensor strip. This device provides a rapid, efficient, cost-effective, and reliable method for the decentralized, noninvasive salivary analysis of BDNF, enabling broader applications in healthcare, wellness monitoring, and medical diagnostics related to the neurotrophin family of biomarkers.

## Graphical abstract

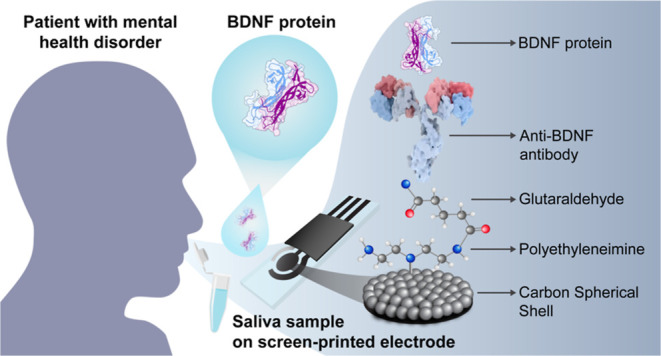

## 1 Introduction

Mood disorders are among the most prevalent, recurrent, and disabling mental illnesses. They can be classified as major depressive disorder, bipolar I disorder, bipolar II disorder, and adjustment disorder with depressive mood. Major depressive disorder (MDD), also known as depression, affects approximately 17% of the global population at some point in their lives, resulting in major social and economic consequences.^1^ Indeed, depression currently affects 280 million people worldwide, according to World Health Organization (WHO) estimates, being a leading cause of disability.^2^ It is different from habitual mood fluctuations and short-lived emotional responses to the challenges of everyday life. Most episodes of depression are classified as moderate or severe and may lead to a great loss of quality of life and productive years.^3^ More than 700,000 people globally die by suicide every year, making it the third leading cause of death in young people aged 15 to 29 globally in 2021 according to WHO.^4^ The pathophysiology of depression is multifactorial and not fully understood. Several pathophysiological systems are implicated, including the immune system, the autonomic nervous system, and primary brain systems such as the monoaminergic brain circuits and the neurotrophic supportive pathway.^5^ A possible biological mechanism was proposed by Duman and co-workers,^6^ referred to as the “neurotrophin hypothesis of depression”. This hypothesis postulated that depression was due to dysfunctional neurogenesis in brain regions responsible for emotion and cognition.^7^ According to this hypothesis, the expression of neuronal growth factors (neurotrophins) is reduced in the face of a stressor.^8^

Neurotrophins (NTs) are signaling proteins responsible for the survival, growth, and development of the mammalian peripheral and central nervous systems.^8,9^ These proteins trigger and regulate neurogenesis, which is the ability to generate new nerve cells from existing neural stem cells.^10^ There are several types of NTs, such as nerve growth factor (BGF), neurotrophin-3 (NT-3), brain-derived neurotrophic factor (BDNF), and NT-4/5.^11^ In particular, BDNF has been known to be expressed in the central and peripheral nervous systems through binding with the tropomyosin kinase B (TrKB) receptor.^9^ The regulated synthesis and secretion of BDNF play a role in learning and memory, neuronal growth, synaptic transmission, and neurotransmitter plasticity.^11^ Biological, pharmacological, and genetic studies have suggested that BDNF is a significant factor involved in major neurological and psychiatric disorders such as Alzheimer’s,^10^ Parkinson’s, Huntington’s, amyotrophic lateral sclerosis, stroke, bipolar disorder, depression, and stress.^9,12,13^ The neurotrophic hypothesis of depression is based on the correlation between lower BDNF levels and a higher frequency of depression, depressive symptomatology, neuronal loss, and cortical atrophy; also, the restoration of the effect of BDNF is linked to antidepressants.^14^ Thus, BDNF is a strong biomarker candidate for depression, which can be exploited for drug screening, therapeutics, and clinical treatments.

BDNF has been analyzed in various neuronal cells using enzyme-linked immunosorbent assay (ELISA),^15,16^ electrochemiluminescence^17^ and fluorescence^18^ measurements, and high-performance liquid chromatography (HPLC).^19^ Though useful, these techniques are time-consuming, requiring large sample volumes, inadequate for on-site analysis, and unsuitable for point-of-care testing.^20,21^ Moreover, the analysis time is long, done in specialized laboratories requiring qualified personnel.^22^ Alternatively, electrochemical methods are promising due to their robustness, cost-effectiveness, and miniaturization capabilities.^23^ Biosensors can be useful for early diagnosis of BDNF and monitoring treatment, particularly for cases in which the disease is typically diagnosed in advanced stages.^22,24,25^ Electrochemical impedance spectroscopy (EIS) has been widely employed in (bio)­chemical sensing and medical diagnostics as a versatile tool for screening disease biomarkers including nucleic acids (DNA and RNA),^26,27^ proteins,^28^ and viruses.^29^ Impedance-based biosensors are useful for an early, accurate detection of biomarkers in noninvasive human biofluids (urine, tears, saliva, and sweat).^30,31^

Here, we report a methodology for convenient, on-site, reliable, and accurate self-testing of salivary BDNF with a disposable immunosensing platform. Salivary biofluid was chosen due to the easy accessibility (it does not require skin puncture) of a noninvasive source, originating from salivary glands, microorganisms, or blood permeation via transcellular and paracellular pathways.^32^

## 2 Experimental Section

All of the specifications for the materials employed in the sensors and in the measurements are given in the Supporting Information.

### 2.1 Preparation of Carbon Spherical Shells (CSSs)

The carbon spherical shells (CSSs) were synthesized following our earlier research.^33–35^ Briefly, 6.5 g of glucose was put in a polytetrafluoroethylene (PTFE) recipient, followed by the addition of 72 mL of high-purity water, and kept under vigorous agitation for 15 min. The reaction system was transferred to a sealed autoclave vessel (100 mL of capacity) and submitted to a hydrothermal treatment using a microcontroller (Microtube, FESORDN, Brazil). The equipment was set to reach 180 °C with a heating ramp of 5 °C min^–1^ and stay there for 5 h before automatically turning off and cooling at room temperature.^33^ The CSS was separated by centrifugation at 12,000 rpm (relative centrifugal force corresponding to 183,881 m s^–2^ or 18,750 × *g*-force) for 30 min. The precipitate was dispersed in 30 mL of ultrapure water (resistivity >18 MΩ cm) followed by a centrifugation step; this procedure was repeated in three cycles. The precipitate was redispersed in 30 mL of ethanol and centrifuged at 12,000 rpm for three cycles. Finally, the material was dried at 90 °C overnight in the oven.^33,34^

### 2.2 Design and Fabrication of the Immunosensor Strip

The flexible sensor (2.5 × 1 cm) was designed via AutoCAD software and then ordered for fabrication of a customized polyester screen (77-mesh, 45 × 35 cm). The pattern has a working electrode (WE) with a geometric area of 12.56 mm^2^.^34,36^ First, the polyester sheet (10 cm × 15 cm) was cleaned using isopropanol and manually aligned below the polyester screen.^34^ Then, the carbon paste was spread onto the screen followed by manual transfer for the substrate using a polyurethane squeegee with a hardness value of 75 Shore and cured at 90 °C for 30 min. The reference electrode layer was made with Ag/AgCl ink and dried at 90 °C for 30 min. Lastly, the isolation layer was created with nail polish to delimit the sensing area. The nail polish was composed by butyl acetate, ethyl acetate, nitrocellulose, isopropyl alcohol, tosylamide/epoxy resin, acetyl tributyl citrate, diacetone alcohol, caprylic/capric triglyceride, stearalkonium hectorite, silica dimethyl silylate, alcohol, oil, seed oil, seed extract, ethylcellulose, flower extract, tocopherol, and malic acid.

### 2.3 Preparation of the BDNF Immunosensor Strip

The flexible devices were submitted to an electrochemical treatment with 0.5 mol L^–1^ sulfuric acid solution to eliminate nonconductive compounds from the printing process. Briefly, 200 μL of H_2_SO_4_ was added to the sensing area, and cyclic voltammetry measurements were performed over the potential range from −2.5 to +2.5 V (two cycles) at 100 mV s^–1^ scan rate. The sensor was rinsed with ultrapure water, and the edges were dried with toilet paper.^37^ Then, 6 μL of CSS suspension (1 mg mL^–1^) was cast on the working electrode (WE) and kept at room temperature (RT) 25 °C until dry (∼3 h). A 6 μL aliquot of polyethylenimine (PEI) (1 mg mL^–1^) was uniformly dropped and allowed to dry at 23 °C overnight. 20 μL of glutaraldehyde solution (1 wt % in water) was used to bond with the amine groups in the PEI layer and incubated at RT 25 °C for 1 h. The devices were gently washed with ultrapure water to remove the aldehyde excess. Then, the anti-BDNF antibodies (10 μL) were immobilized in a humidity chamber at 5 °C, followed by rinsing with phosphate buffer (pH = 7.0, 0.1 M) to remove the unbound antibody from the detection area. The immobilization time and anti-BDNF concentration were optimized in the ranges of 1 to 12 h and 0.5 to 2.0 μg L^–1^, respectively. The sensor surface was blocked with 1.0 M ethanolamine solution for 1 h to prevent nonspecific adsorption. The BDNF immunosensor strip was stored at 4 °C until the use. Before the assembly process, the immunosensor was gently washed with phosphate buffer solution (pH = 7.0, 0.1 M) for 3 times to remove unloaded chemicals. The schematic in Figure 1 summarizes the steps in preparation of the BDNF immunosensor strip.

**Figure 1:**
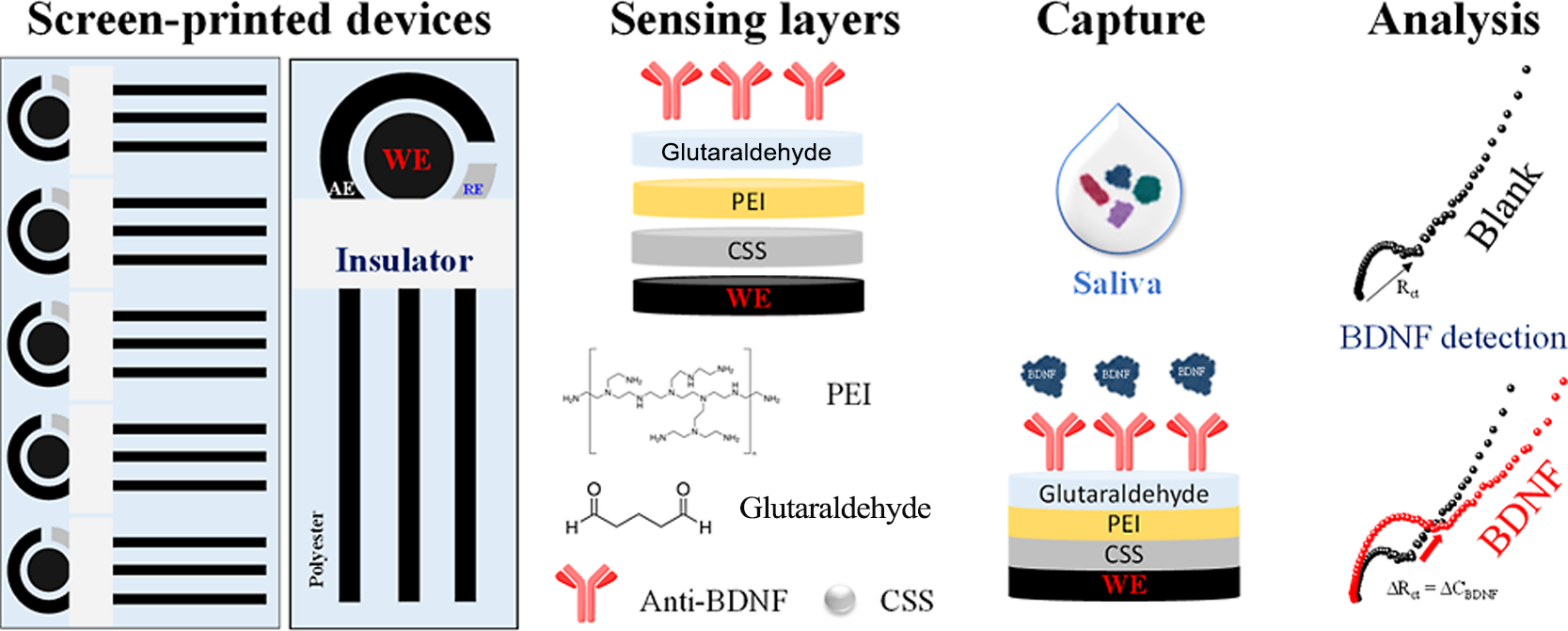
Concept and schematic representation of a disposable immunosensor strip for decentralized analysis of salivary BDNF, highlighting the procedure to detect salivary BDNF including saliva sampling, testing, and analysis on a device.

### 2.4 Electrochemical Measurements

The immunosensor was incubated with 20 μL of BDNF solution (between 1.0 × 10^–20^ and 1.0 × 10^–10^ g mL^–1^) for 30 min at room temperature; then, the immunosensors were rinsed with phosphate buffer solution to remove the unbound BDNF proteins. Finally, 150 μL of Fe­(CN)_6_^3–/4–^ (5 mM) prepared in 0.1 M phosphate buffer solution was added to the BDNF immunosensor strip, and an EIS measurement was recorded. The working frequency was between 0.1 Hz and 10,000 kHz under open circuit potential with 10 mV of amplitude. Following each successive addition of BDNF, the impedimetric responses were monitored to track the changes in the *R*_ct_ values with respect to the concentration. The analytical signal was obtained from the difference between *R*_ct_ in the presence and absence of the BNDF antigen (Δ*R*_ct_ = *R*_ct BDNF_ – *R*_ct ethanolamine_). The semicircle in the Nyquist plot corresponds to the charge-transfer resistance, *R*_ct_.^31^

### 2.5 BDNF Analyses in Human Saliva Using the Immunosensor Strips

The BDNF determination in saliva samples was conducted in compliance with the protocol approved by the ethical committee from Brazil (projects 54796721.8.0000.5422 and 60626222.5.0000.5422). The human saliva samples were provided by a 30 year-old female volunteer. To prepare the samples, 100 μL of raw saliva was diluted in 1900 μL of phosphate buffer solution and then doped with BDNF standard solutions. The immunosensor was incubated with 20 μL of doped saliva (between 1.0 × 10^–20^ and 1.0 × 10^–10^ g mL^–1^) for 30 min at room temperature; then, the immunosensors were rinsed with phosphate buffer solution to remove the unbound BDNF proteins. Subsequently, 150 μL of Fe­(CN)_6_^3–/4–^ (5 mM) prepared in 0.1 M phosphate buffer solution was added to the BDNF immunosensor strip, and an EIS measurement was recorded. The working frequency was between 0.1 Hz and 10,000 kHz under open circuit potential with 10 mV of amplitude. As in the experiments described in the previous subsection, following each successive addition of doped saliva, the impedimetric responses were monitored to track the changes in *R*_ct_ values with respect to the concentration.

## 3 Results and Discussion

### 3.1 Working Principle and Reagent Layer Configuration of Disposable Immunosensing Strips for BDNF Detection

We demonstrate noninvasive self-testing to monitor biomarker levels for mental disorders using an inexpensive and disposable immunosensing strip, which may allow for a comfortable, fast, and decentralized noninvasive analysis of BDNF. The concept and schematic representation of the immunosensing strip in Figure 1 highlight the method for measuring salivary BDNF. It includes saliva sampling, testing, and analysis on an immunosensor strip containing a functionalized working electrode (WE), a bare carbon auxiliary electrode (AE), and a silver/silver chloride reference (RE) electrode screen-printed on a polyester film (PE) support. The working electrode (WE) was modified with CSSs, followed by functionalization with a polymeric layer of PEI and glutaraldehyde for enhancing sensitivity and acting as a matrix to attach recognition biomolecules. The BDNF-specific capture antibody (anti-BDNF) was immobilized by drop casting, followed by an ethanolamine reagent layer to block active sites, thus avoiding nonspecific interactions at the sensing surface. A small volume of 100 μL collected saliva is dropped on the immnunosensor’s sensing zone without any sample pretreatment. The disposable immunosensor strip can be connected to a portable potentiostat, offering fast (within 35 min) measurements of salivary BDNF. The results can be displayed in real time on a mobile device through wireless communication (Bluetooth).^32,38^ The BDNF detection occurs via the formation of antibody–antigen immunocomplexes, which increases the charge-transfer resistance at the sensing surface. This increase is captured with electrochemical impedance spectroscopy, as illustrated in Figure 2. The disposable immunosensor strip offers a large dynamic range from 1.0 × 10^–20^ to 1.0 × 10^–10^ g mL^–1^ (covering both normal and elevated BDNF levels in human saliva), high selectivity, stability, and reproducibility.

**Figure 2:**
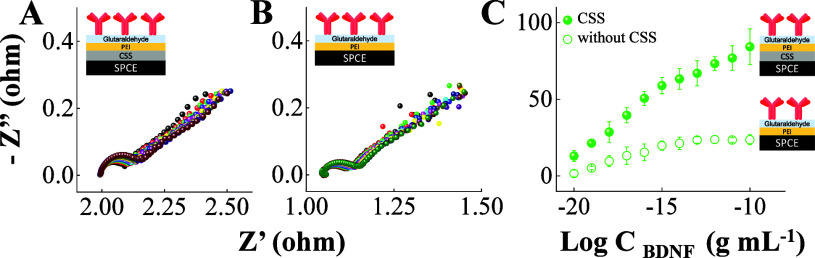
Effects of the CSS on matrix layer configuration to detect BDNF. Nyquist responses of the SPCE/CSS/PEI/Glu/Ab/Et strip in (A), the SPCE/PEI/Glu/Ab/Et (without the CSS) strip in (B), and the corresponding BDNF calibration plots in (C) to successive levels of BDNF detection ranging from 1.0 × 10^–20^ to 1.0 × 10^–10^ g mL^–1^ in 0.1 M phosphate buffer solution.

The strategic employment of distinct layers is crucial for tracking the BDNF biomarker with a high analytical performance. The BDNF detection was evaluated for two different layers on the WE surface. In the first strategy in Figure 2A, we employed a CSS layer, followed by deposition of a polymeric layer of PEI and glutaraldehyde. The increased Nyquist response in Figure 2A is a consequence of the antigen capture by antibodies immobilized on the sensing surface. The sensitivity and dynamic range of detection decreased in Figure 2B when the polymeric layer containing only PEI and glutaraldehyde (without the CSS) was used as a matrix to attach antibodies. After the BDNF target detection step, *R*_ct_ increased, as shown in Figure 2, suggesting immunocomplex formation and the consequent blockage of the electroactive area. The immunosensor performance in Figure 2A was improved by CSS addition to the PEI/glutaraldehyde matrix, enhancing the recorded Nyquist signals, the sensitivity, and the linear range of BDNF detection. This improvement can be assigned to the judicious combination of distinct materials, which are used to confine the biomolecules and enhance the sensitivity and dynamic range of analytical systems.^32^ The synergistic combination of the CSS, PEI, and glutaraldehyde allowed a sensitive, rapid, and selective immunosensor strip to detect BDNF with highly reproducible and stable Nyquist profiles. PEI and glutaraldehyde served as a matrix enabling the stable attachment of anti-BDNF antibodies via imine-type covalent bonding (CH=N) and minimizing the biofouling effect on the sensing surface.^39^ The strategy depicted in Figures 1 and 2A was adopted for subsequent measurements.

An explanation for the improved sensing performance in Figure 2 can be drawn from the characterization results for CSSs in Figure 3. The Raman spectrum in Figure 3A has two well-defined peaks at 1341 and 1577 cm^–1^ related to the defects band of the D band and vibration of the sp^2^ carbon of the G band. The weak D band and intense G band reveal graphite carbon with few sp^3^ defects.^40^ A large peak at ∼2600–2900 cm^–1^ consisting of two low-intensity peaks at ∼2668 cm^–1^ and ∼2888 cm^–1^ is assigned to the 2D and D + D′ bands.^41^ The vibration bands in the Fourier transform infrared spectroscopy (FTIR) spectrum in Figure 3B indicate that the CSS surface is rich in hydrophilic carbonyl and hydroxyl groups. They include O–H stretching vibration from hydroxyls or carboxylic groups at 3388 cm^–1^ or from water (considering the broadness of the peak) remaining from washing and synthesis steps; C–H stretching vibrations of aliphatic groups at 2923 cm^–1^; C=O from the carbonyl group and C=C stretching vibrations at 1700 and 1620 cm^–1^; and −C–OH stretching and −OH bending vibrations from hydroxyl groups at 1000–1400 cm^–1^. Bands in the range around 800 cm^–1^ can be assigned to out-of-plane C–H bond vibrations.^42^ The broad peak at 21.6° in the X-ray diffraction (XRD) pattern (Figure 3C) of the CSS points to the typical amorphous nature of some carbon materials.^42^ The X-ray photoelectron spectroscopy (XPS) spectrum survey in Figure S1 with binding energies at 284.5 and 532.5 eV indicates that the CSS contains 81.3% carbon and 18.7% oxygen. The deconvolution of the high-resolution XPS spectra for C 1s in Figure 3D resulted in five components with binding energies at 283.8 and 284.5 eV (graphitic carbon) assigned to single and double bonds at 285.4, 286.4, and 287.9 eV for C–OH, C=O, and HO–C=O bonds, respectively. The high-energy subpeak at 288.8 eV is assigned to the π → π* interband transition, also named the plasmon excitation satellite peak.^43^ The O 1s high-resolution spectrum in Figure 3E has three components with binding energies at 531.3 and 532.5 eV assigned to C–OH, C–O–C, and C=O and at 533.6 eV related to the water molecules chemisorbed on the CSS surface. Hence, XPS shows a CSS surface rich in oxygenated groups. Transmission electron microscopy (TEM) and Field Emission Gun-Scanning Electron Microscopy (FEG-SEM) images in Figure 3F,G demonstrate a uniform spherical morphology of the CSS with an average diameter of 175 ± 25 nm, with the FEG-SEM image showing densely packed CSSs with particles in interfacial contact forming a homogeneous structure. The three-dimensional overlapping of interconnected CSSs creates a dense and rough surface with a high surface area contributing to the efficient immobilization of biomolecules. The energy-dispersive X-ray spectroscopy (EDS) mappings in Figure 3H,I reveal the elemental composition for a packed CSS with the amount of carbon and oxygen matching the spherical structure with C and O elements well-distributed on the CSS surface. The EDS spectrum in Figure S2 depicts only carbon and oxygen, in addition to gold signals from the sample preparation step for SEM analysis, thus demonstrating the absence of contaminants.

**Figure 3:**
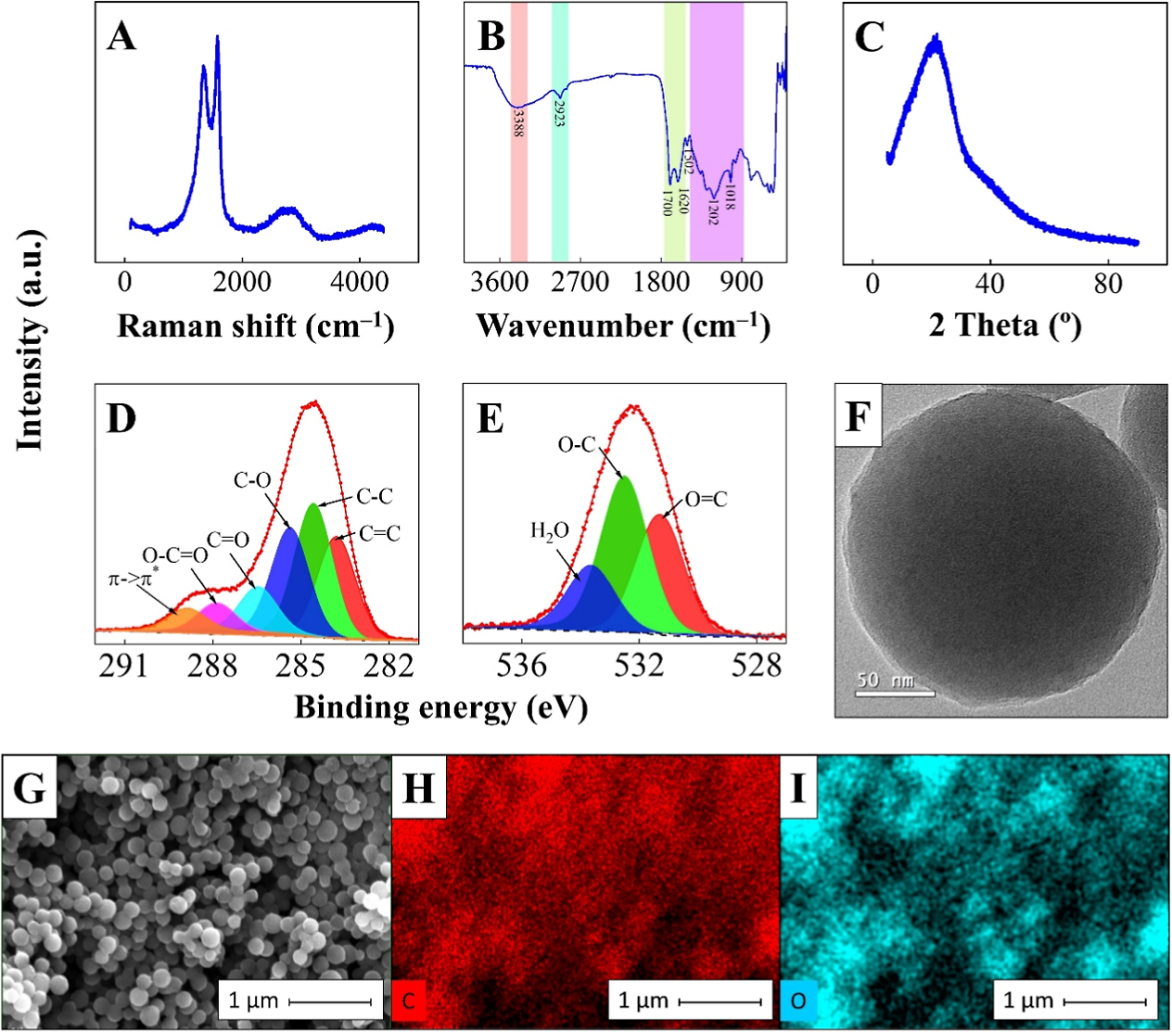
Features of the CSS. (A) Raman spectrum. (B) ATR-FTIR spectrum. (C) Powder XRD pattern. High-resolution X-ray photoelectron spectra for C 1s in panel (D) and for O 1s in panel (E). Black dots and red lines refer to the experimental data, and colored peaks represent the fitted spectra. (F) TEM image of the CSS. (G) FEG-SEM image and corresponding elemental mapping data of carbon (H) and oxygen (I) for the CSS.

An efficient BDNF immunosensing platform requires a selection of a suitable time for biorecognition molecule immobilization and anti-BDNF concentration to achieve optimized analytical performance.^38^ The impact of the immobilization time and anti-BDNF concentration is shown in the analytical curves in Figure 4. The responses increased significantly with the antibody concentration from 0.5 to 2.0 μg L^–1^. The highest linear detection range between 1.0 × 10^–20^ and 1.0 × 10^–12^ g mL^–1^ and highest sensitivity were obtained with the 2.0 μg L^–1^ anti-BDNF concentration. As expected, higher amounts of antibodies led to an increasing availability of active sites at the immunosensor strip surface to capture BDNF. The analytical responses in Figure 4B increased with biomolecule immobilization time from 1 to 2 h, after which they remained practically the same. Therefore, 2 h suffices for a complete biomolecule attachment on the immunosensor strip at 2.0 μg L^–1^ anti-BDNF. The shorter immobilization time with improved analytical performance is excellent for fast assembly and detection.

**Figure 4:**
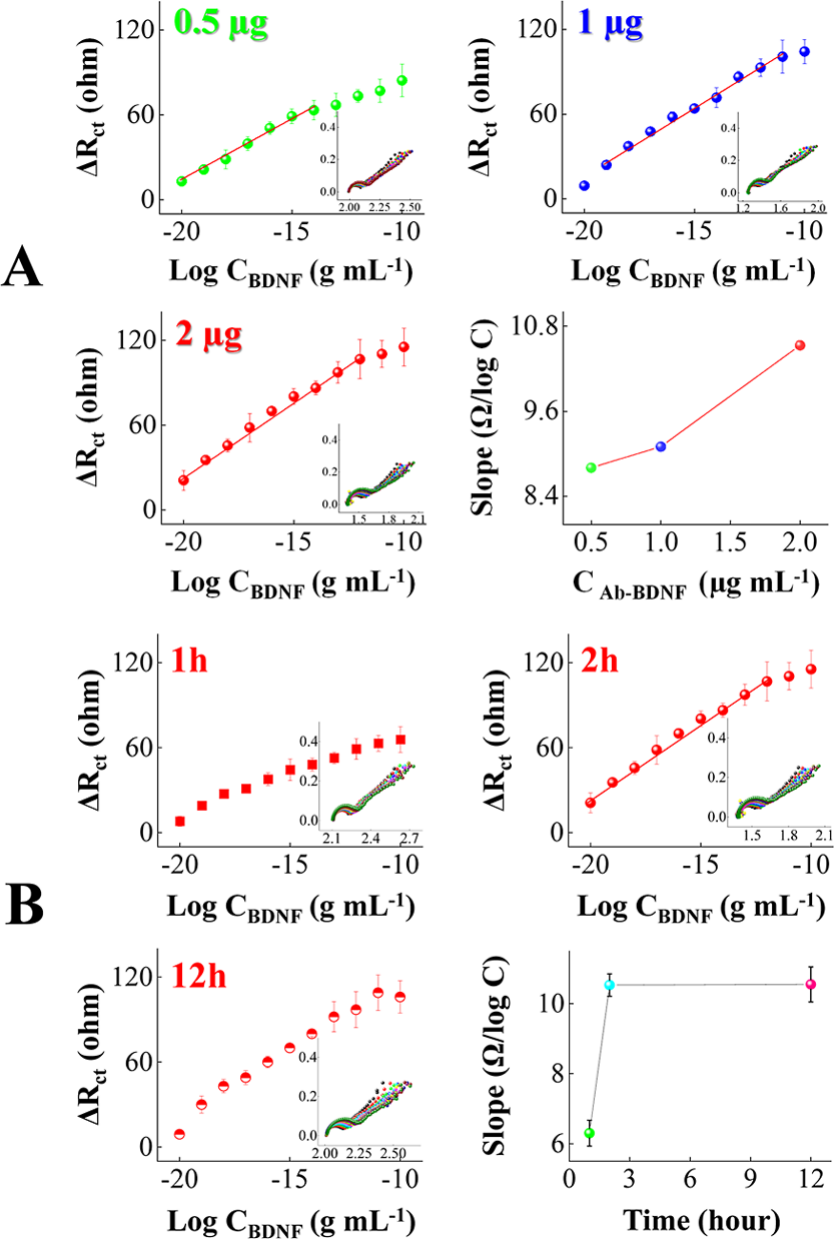
Impact of biorecognition molecule immobilization time and anti-BDNF concentration to detect BDNF. Nyquist responses of immunosensing strips with BDNF concentrations ranging between 1.0 × 10^–20^ and 1.0 × 10^–10^ g mL^–1^ in 0.1 M phosphate buffer solution with corresponding calibration plots in different antibody amounts immobilized at the sensor strip surface varying from 0.5 to 2.0 μg L^–1^ (A) and in different immobilization periods (B).

### 3.2 Electroanalytical Sensing Performance of BDNF Detection on Disposable Immunosensor Strips

Under optimal reagent layer configuration including a biological recognition element, the analytical performance of the disposable immunosensor strip was assessed as shown in Figure 4B (top right side) over a wide range of BDNF levels in 0.1 M phosphate buffer solution. The disposable immunosensor strip had a well-defined Nyquist response to successive detections of BDNF ranging from 1.0 × 10^–20^ to 1.0 × 10^–10^ g mL^–1^, which correspond to normal and elevated BDNF levels in human saliva. The measured response is fitted by the calibration curve described by the equation Δ*R*_ct_ = 235.3 + 10.5 log *C*_BDNF_ (*R* ^2^ = 0.99) with a sensitivity of 8.35 Ω g^–1^ mL cm^–2^ for BDNF levels between 1.0 × 10^–20^ and 1.0 × 10^–10^ g mL^–1^. The lowest detectable concentration of 1.0 × 10^–20^ g mL^–1^ confirms the suitability of the immunosensor strip to monitor ultralow levels of BDNF. The high detection performance of the immunosensor strip allows for detecting BDNF at a wide range of levels, including high and low concentrations from healthy and diseased patients.^44^ This versatility extends the device utility for disease management of patients including those with mental disorder to general individuals who are at risk or those who want to proactively maintain healthy BDNF levels.^38^

With the optimal configuration of the disposable immunosensing system established, the in vitro performance evaluation of the BDNF immunosensor strips was conducted as shown in Figure 5 through relevant parameters including reproducibility, storage stability, and selectivity. The reproducibility study in Figure 5A was conducted using three BDNF immunosensors in which the slopes from analytical curves were obtained in triplicate in the range from 1.0 × 10^–20^ to 1.0 × 10^–10^ g mL^–1^. The relative standard deviation (RSD) of 5.32% (*n* = 3) indicated excellent reproducibility. The BDNF immunosensors retained 90% of their initial response after 7 days when stored at 4 °C, with a small decrease in performance after 30 days, as shown in Figure 5B. This can be considered a satisfactory storage capacity for a cost-effective immunosensor strip device. The selectivity was assessed in Figure 5C with measurements taken in the presence of probable coexisting human salivary compounds. The Nyquist responses in Figure 5D had negligible impacts on the BDNF detection signal in the presence of glucose, paracetamol, BSA, uric acid, NaCl, ascorbic acid, and lactate, with changes in Δ*R*_ct_ of −3 Ω, −1 Ω, −10 Ω, −2 Ω, −1 Ω, −9 Ω, and −11 Ω, respectively. A minimal increase in Δ*R*_ct_ was observed for urea (5 Ω) and SARS-CoV-2 S protein (5 Ω), whereas the Δ*R*_ct_ for 1.0 × 10^–17^ g mL^–1^ BDNF protein was 60 Ω. Overall, the main constituents of human saliva have a negligible effect on the performance of the BDNF immunosensing system.

**Figure 5:**
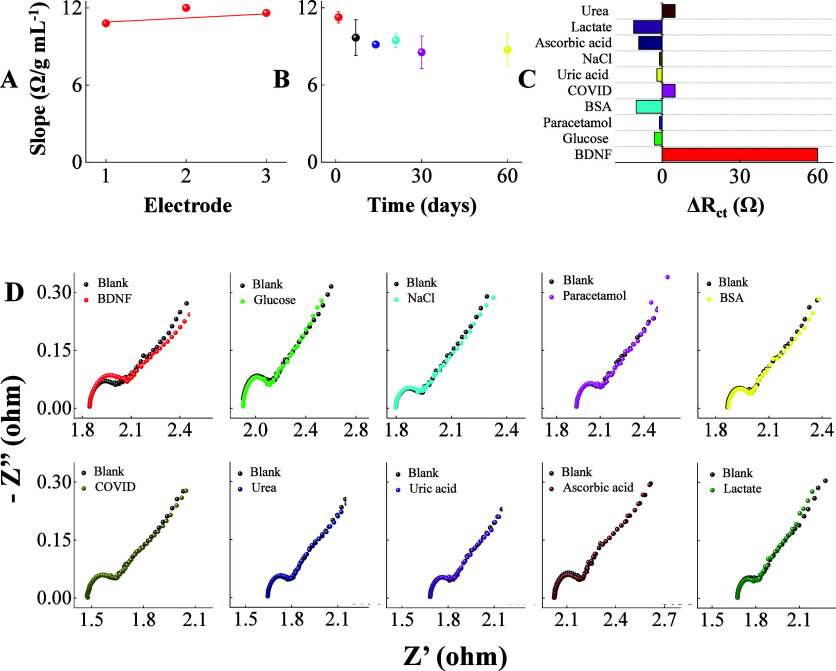
Performance of the BDNF immunosensor strips in a 0.1 M phosphate buffer solution. (A) Reproducibility analysis with different devices. Slope responses from analytical curves were obtained using three immunosensor strips for BDNF detection ranging from 1.0 × 10^–20^ to 1.0 × 10^–10^ g mL^–1^ in 0.1 M phosphate buffer solution. (B) Long-term storage stability study throughout 60 days. Slope responses from analytical curves were obtained in triplicate with immunosensor strips for BDNF detection from 1.0 × 10^–20^ to 1.0 × 10^–10^ g mL^–1^. (C) In vitro selectivity analysis against potential interfering molecules. (D) Nyquist response of the immunosensor strips on potential interferents molecules found in human salivary samples such as glucose, NaCl, paracetamol, BSA, SARS-CoV-2 S protein, urea, uric acid, ascorbic acid, and lactate in 0.1 M phosphate buffer. Nyquist plots were recorded in 5.0 mM K_4_[Fe­(CN)_6_] + K_3_[Fe­(CN)_6_] in 0.1 M phosphate buffer. The interferent concentration and BDNF target were fixed at 1 μg mL^–1^ and 1.0 × 10^–17^ g mL^–1^, respectively.

### 3.3 Detection of BDNF in Human Saliva with the Disposable Immunosensor Strip

Figure 6 shows the BDNF analysis in human saliva with well-defined Nyquist responses for concentrations ranging from 1.0 × 10^–20^ to 1.0 × 10^–10^ g mL^–1^. The change in charge transfer resistance (Δ*R*_ct_ = *R*_ct BDNF_ – *R*_ct ethanolamine_) varies linearly with log *C*_BDNF_ according to 5.3 log *C*_BDNF_ + 121 (*R* ^2^ > 0.98). The lowest detectable concentration of 1.0 × 10^–20^ g mL^–1^ in human saliva indicates the suitability of the BDNF biosensing method for salivary tracking. This reflects the effectivity of the sustainable and cost-effective matrix used in the BDNF immunosensor strip preparation to avoid adsorption effects and related electrode biofouling in this complex human biological fluid.^45^

**Figure 6:**
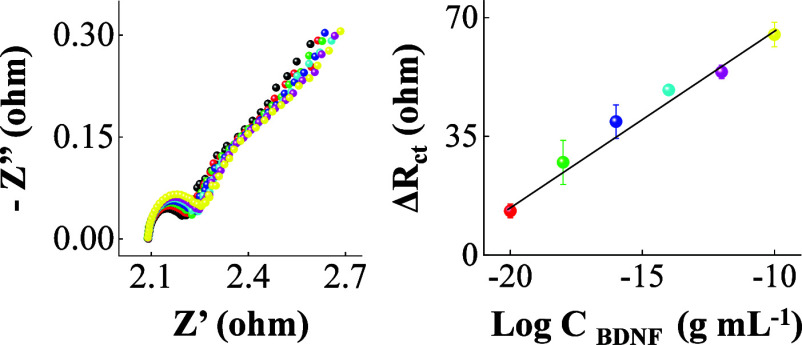
Salivary sample analysis with increased BDNF levels. Nyquist responses of the BDNF immunosensor strip in concentrations ranging from 1.0 × 10^–20^ to 1.0 × 10^–10^ g mL^–1^ with the corresponding BDNF analytical curve. Each Nyquist plot corresponds to a concentration of BDNF.

The interpolation method was applied to estimate BDNF levels, avoiding possible variabilities in the slope value of the calibration plots constructed from saliva collected from a healthy patient. Table 1 summarizes the excellent recoveries of 91 ± 6% and 107 ± 1% found in human saliva samples containing BDNF using the immunosensor strip.

**Table 1:** Determination of BDNF in Human Saliva Non-invasive Samples with the Disposable Immunosensor Strip

sample	added (g mL^–1^)	found (g mL^–1^)	recovery %
saliva 1	10 × 10^–19^	9.1 × 10^–19^	91 ± 6.1
saliva 2	1.0 × 10^–16^	1.07 × 10^–16^	107 ± 1.0

Table 2 compares the sensing layer, analytical performance, disposability, miniaturization, and biological sample of the disposable immunosensor strip with other devices reported in the literature. The sensitivity is superior in terms of the lowest detectable values (LDV) and the dynamic detection range (1.0 × 10^–20^ to 1.0 × 10^–10^ g mL^–1^) for BDNF. The broad screening range comprising low concentrations is relevant and an advantage because mental disorders cause reductions in salivary BDNF levels that are highly variable and affected by diverse factors, including emotional condition, gender, and stress level of patients.^46,47^ The superior analytical performance allows rapid, on-site, decentralized, and continuous biochemical assays exhibiting significant advantages in preliminarily alerting to potential abnormalities and in personalized self-monitoring healthy individuals and patients in real time.^44^ Another important advantage is the simple, easy, safe, inexpensive (US$ 2.19 per device considering only consumables and excluding manufacturer time, machine time, and EIS machinery), and friendly sensing platform to detect BDNF in human saliva employing CSSs made of glucose and water precursors, PEI and glutaraldehyde. Other devices in the literature contain metallic nanomaterials and hazardous materials, with complex experimental steps for synthesis and sensor preparation requiring toxic chemicals producing large amounts of toxic waste. Unlike the studies listed in the table, our disposable device offers a convenient, comfortable, and pleasant method to track BDNF levels in human saliva noninvasive samples.

**Table 2:** Comparison of the Analytical and Operational Features of Immunosensors Used to Detect BDNF^a^

Sensing layer	Disposable miniaturized	Invasive analysis	Sample	Linear range (pg/mL)	LDV (pg/mL)	Ref
AuNPs/NG-PANI/ITO-PET	No	Yes	Mice serum	0.8–400	0.8	^48^
SPCE/AuNPs/pTTBA-anti-BDNF	Yes	Yes	Cancer cells	4.0–600.0	4.0	^9^
SPEAu/PmPD	No/Yes	Yes	Serum	10–40	10	^49^
IME-anti-BDNF	Yes	Yes	Mice hippocampus	0.01–10,000	0.01	^50^
PSPS-Au wrinkles	No	Yes	Plasma	100–2000	100	^51^
SPE/CSS/PEI-Glu-anti-BDNF	Yes	No	Saliva	1.0 × 10^–8^ to 100	1.0 × 10^–8^	**This work**

a ITO-PET: indium tin oxide-coated polyethylene terephthalate, NG-PANI: N-doped graphene-polyaniline, (AuNPs): gold nanoparticles, PmPD: *meta*-phenylenediamine, SPEAu: commercial gold electrode, Pttba: 2,2:5, terthiophene-3-(*p*-benzoic acid), IME interdigitated microelectrode, PSPS prestrained polystyrene, and Au wrinkles: micro-/nanoscale wrinkles within the gold film.

### 3.4 Environmental Impact Using Greenness and Blueness Evaluation of the BDNF Detection Method with the Immunosensor Strip

The greenness of detecting BDNF using a disposable immunosensor strip was evaluated through two distinct tools: the Analytical Eco-Scale (AES)^52^ and the Analytical Greenness Metric Approach (AGREE).^53^ AES relies on a semiquantitative analysis to assess greenness considering penalty points (PP) of four factors that negatively impact the environment: the quantity and type of chemicals used, occupational hazards, waste generation, and energy consumption.^52^ By totaling the penalty points, AES is estimated by AES = 100 – PP, with scores above 75 indicating excellent environmental friendliness, scores above 50 representing acceptable green analysis, and scores below 50 indicating inadequate environmental friendliness.^52^ The AGREE metric tool employs the 12 Green Analytical Chemistry (GAC) principles to provide a visual representation in the form of a colored pictogram: green, yellow, or red, assigned for excellent, medium, and bad environmental impact, respectively.^53,54^ A numerical score is given to each criterion, and the overall score is listed in the middle of a clock-shaped pictogram ranging from 0 to 1, with 1 signifying the most environmentally friendly option.^54^ The AES score of 76 in Table 3 is consistent with the AGREE pictogram depicted in Figure 7A for the disposable immunosensor strip, with a score estimated to be 0.75. This indicates a sustainable and green analytical method to detect BDNF.^52,53^

**Table 3:** Penalty points to Estimate the Analytical Eco-Scale for BDNF Detection Using Disposable Immunosensor Strips

	Amount	Penalty points
Chemical		
Sodium phosphate dibasic	<10 mL (g)	0
Sodium phosphate monobasic	<10 mL (g)	0
Glucose	<10 mL (g)	0
Sulfuric acid	<10 mL (g)	2
Ink	<10 mL (g)	1
Ethanol	<10 mL (g)	2
CSS	<10 mL (g)	0
Ethanolamine	<10 mL (g)	4
Glutaraldehyde solution	<10 mL (g)	8
Polyethylenimine branched	<10 mL (g)	2
Anti BDNF	<10 mL (g)	0
Potassium hexacyanoferrate	<10 mL (g)	2
Potassium ferricyanide(III)	<10 mL (g)	2
**Total of penalty points of reagents**		**23**
Instruments		
Energy	⩽0.1 kWh per sample	0
Centrifuge	⩽0.1 kWh per sample	0
Oven	⩽0.1 kWh per sample	0
Occupational hazard	Analytical process hermetization	0
Waste	⩽1 mL (g)	
	No treatment	1
**Total of penalty points for instruments**		**1**
∑ **penalty** **points**		24
**Analytical** E**co-Scale**	100–24	76

**Figure 7:**
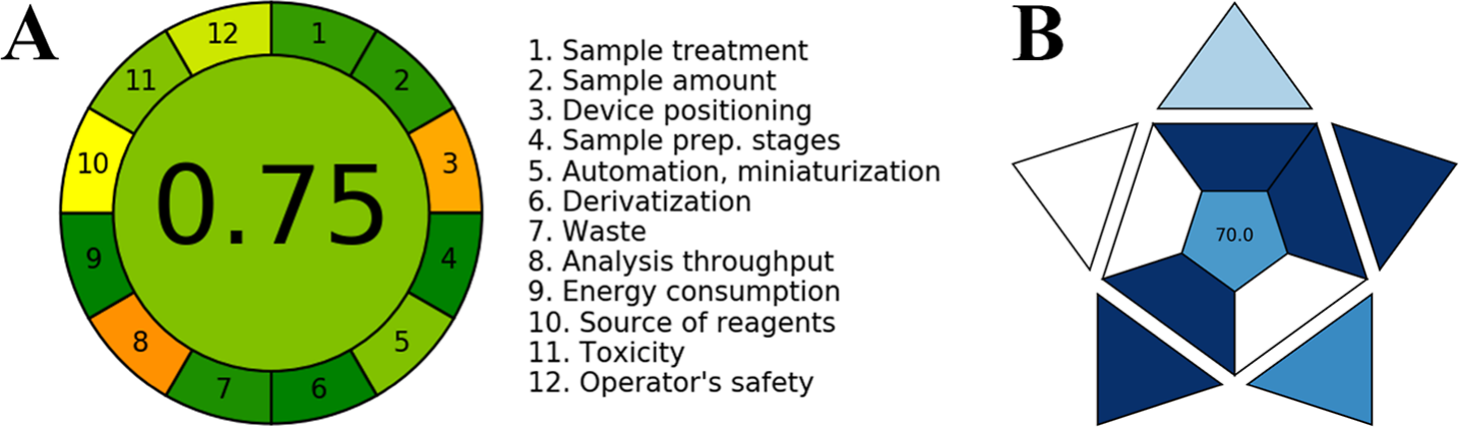
Greenness and blueness assessment of the electroanalytical method using a BDNF immunosensor strip. (A) Agree metrics and (B) blue applicability grade index (BAGI) pictograms for the electroanalytical method using an immunosensor strip to detect BDNF in human saliva samples.

The Blue Applicability Grade Index (BAGI) was employed as a complementary tool to the well-established green metric tools for a manifold evaluation of the practicality (blueness) of the analytical methodologies.^54–56^ BAGI evaluates criteria aligned with the principles of environmental sustainability that determine the practicality of an analytical method, i.e., type of analysis, number of analytes, sample preparation, sample volume, sample throughput and simultaneous sample preparation, reagents and materials, instrumentation and automation degree, the fitness for purpose, and the automation degree.^54–56^ Using the available software in the open-source application mostwiedzy.pl/bagi, an asteroid pictogram is produced along with the respective score ranging between 25 and 100, which is recommended to be higher than 60.0 to be considered an applicable analytical method.^55,56^ The color scale of dark blue, blue, light blue, and white indicates high, medium, low, and no compliance with the established criteria, respectively.^56^ The worst method assessment in terms of applicability is indicated by a score of 25.0, while a score value of 100 indicates excellent method applicability.^55,56^ The BAGI pictogram with score of 70 depicted in Figure 7B is higher than 60.0, indicating that our electroanalytical method using immunosensor strip to detect BDNF in human saliva samples is an applicable analytical method.^54–56^

## 4 Conclusions

We reported an inexpensive and disposable immunosensor strip for the noninvasive salivary analysis of brain-derived neurotrophic factor (BDNF). A CSS layer deposited on a screen-printed carbon electrode was coated with an ultrathin film of PEI and glutaraldehyde, serving as a matrix for the immobilization of the anti-BDNF biological recognition element. The analytical performance of the BDNF immunosensor strip was characterized by in vitro measurements, demonstrating fast analysis time, robustness, reproducibility, good tolerance to potential interfering substances, and a broad dynamic range, with a limit of detection as low as 1.0 × 10^–20^ g mL^–1^—suitable for BDNF detection in human saliva. Saliva samples were easily collected using a simple system with a Falcon tube and analyzed directly on the immunosensor strips without the need for centrifugation. No interference from other salivary constituents was observed. This disposable salivary BDNF detection technology may be extended to multiplexed assays or integrated into multianalyte salivary strips for applications in personalized medicine and wellness, including early monitoring of health status and mental disorders. The combination of the disposable BDNF immunosensor strip with rapid, decentralized saliva analysis presents significant promise for a wide range of medical applications.

## Supplementary Material



## Data Availability

All data needed to evaluate the conclusions in this study are present in the paper and/or the Supporting Information. Additional data related to this paper may be requested from the authors.

## Notes

XPS survey spectrum of CSSs and EDS spectrum of CSSs (PDF)
