# Supplementary material for: Low-Cost, Disposable Biosensor for Detection of the Brain-Derived Neurotrophic Factor Biomarker in Noninvasively Collected Saliva toward Diagnosis of Mental Disorders
Source: ACS Polym Au. 2025 Aug 1;5(4):420–31. doi: 10.1021/acspolymersau.5c00038 (PMC12355618; doi:10.1021/acspolymersau.5c00038)
Supplement: Supplementary file 1 [file lg5c00038_si_001.pdf]

Supporting Information

Low-cost, disposable biosensor for detection of brain-derived neurotrophic factor biomarker in non-invasively collected saliva toward diagnosis of mental disorders

Nathalia O. Gomes[a,b], Marcelo L. Calegaro[a], Luiz Henrique C. Mattoso[b], Sergio A. S. Machado[a], Osvaldo N. Oliveira Jr.[c], Paulo A. Raymundo-Pereira[c,*]

[a] *Sao Carlos Institute of Chemistry, University of Sao Paulo, IQSC – USP, 13560-970, Sao Carlos, SP, Brazil*

[b] *Nanotechnology National Laboratory for Agribusiness (LNNA), Embrapa Instrumentation, 13561-206, Sao Carlos, SP, Brazil*

[c] *Sao Carlos Institute of Physics, University of Sao Paulo, IFSC – USP, 13566-590, Sao Carlos, SP, Brazil*

*Corresponding author:

Sao Carlos Institute of Physics, University of Sao Paulo, IFSC – USP, 13566-590, Sao Carlos, SP, Brazil. E-mail address: pauloaugustoraymundopereira@gmail.com (Paulo A. Raymundo-Pereira). Tel.: +55 16 33739825.

Number of pages: 10

Number of tables: 0

Number of figures: 2

Table of content

Figure S1 – XPS survey spectrum of CSS………………………………………………….. 9

Figure S2 – EDS spectrum of CSS………………………………………………........ 10

1. Experimental details

1.1 Chemicals

Potassium chloride (99%), estradiol (98%), uric acid (99%), paracetamol (99%), ascorbic acid (99%), sodium nitrate (99%) sodium phosphate dibasic (98.5%), sodium phosphate monobasic monohydrate (98%) potassium hexacyanoferrate (III) (99%), potassium hexacyanoferrate (II) trihydrate (99%), ethanolamine (>99%), Glucose (99%), polyethylenimine branched (average Mw~25,000; average Mn ~10,000 by GPC; linear formula: $H(NHCH_2CH_2)_nNH_2$. The material dispersity (Mw / Mn) is 25,000 / 10,000 = 2.5), albumin bovine (96%), lactic acid solution (85%), urea (98%), sodium chloride (99%), glutaraldehyde solution (50% wt in water) was purchase from Sigma Aldrich (St. Louis, MO, USA). All solutions were prepared with high-purity water (resistivity > 18 MΩ cm). Carbon conductive ink was acquired from LOCTITE® (423SS E&C). Silver conductive paste was purchased from TICON®, (CODE: 90701, BRAZIL). The copper grids were obtained from Ted Pella®. A commercial transparent

polyester film (PE) substrate (100 μm) was used to print the carbon ink patterns. The antibodies anti-BDNF (HPA056104, 0.05 mg mL^{-1}) and brain derived neutrophic factor human protein (B3795, 10 UG) were acquired from Sigma Aldrich (St. Louis, MO, USA). Anti-BDNF and BDNF working solutions were prepared in 0.1 M phosphate buffer solution (pH ≈ 7) and stored at −20 ºC until use. Phosphate buffer solution was prepared solubilizing sodium phosphate monobasic and sodium phosphate dibasic in water, yielding a 0.1 M solution at pH ≈ 7.0.

1.2 Apparatus

1.2.1 Raman

Raman analysis was made in a Horiba spectrometer, model LabRAM HR evolution, (HORIBA France SAS, Longjumeau, France), with an excitation wavelength of 514 nm and a power of 25 mW.

1.2.2 Fourier transform infrared

Fourier transform infrared (FTIR) experiments with an attenuated total reflectance (ATR) module were performed with a Bruker spectrophotometer (Model Tensor 27) over a wavenumber range between 4000 and 400 cm^{-1}, with a resolution of 4.0 cm^{-1} and 36 scans.

1.2.3 X-ray diffraction

X-ray diffraction (XRD) spectra were taken with a diffractometer D8 Advance (Bruker, USA) using CuKα radiation. The identification and assignment of peaks were performed with Crystallographica Search-Match 2.1.1.1 (Oxford Cryosystems, UK).

1.2.4 X-ray photoelectron spectroscopy

The chemical composition of CSS was evaluated by X-ray photoelectron spectroscopy (XPS) using a spectrometer (ScientaOmicron ESCA+) with a high-performance hemispheric analyzer (EAC2000) with monochromatic Al Kα ($h\nu$ = 1486.6 eV) radiation as an excitation source. The operation pressure in the ultrahigh vacuum chamber (UHV) during the analysis was 1×10^{-9} Pa. The XPS high-resolution spectra were recorded at a constant pass energy of 20 eV with 0.05 eV per step.

1.2.5 Transmission electron microscopy

Transmission electron microscopy (TEM) images were recorded with a JEM 2100 microscope (JEOL, Japan) operating at 200 kV. CSS were ultrasonically suspended in water (1 mg mL^{-1}) for 30 min and dropped on carbon-coated copper grids.

1.2.6 Scanning Electron Microscopy

Field Emission Gun – Scanning Electron Microscopy (FEG-SEM) images were obtained with microscope from Japan Electron Optics Ltd. (JEOL), model: JSM 7200F, equipped with a microanalysis system by energy dispersive X-ray spectroscopy (EDS) from BRUKER Corporation, USA, model: XFLASH 6–60. The samples were fixed to solid supports with a conducting double-faced carbon tape.

1.2.7 Electrochemical analysis

The electrochemical measurements were conducted using a PGSTAT 302 potentiostat from Autolab controlled by NOVA 2.1.5 software in the frequency range between 0.1 Hz and 100 kHz with amplitude of 10 mV and under open circuit potential (OCP) conditions in 0.1 M phosphate

buffer solution solution containing 5.0 mM of $K_3[Fe(CN)_6]/K_4[Fe(CN)_6]$. All measurements were carried out at room temperature 25 °C. The solution was neither stirred nor aerated during the measurements.

1.3 Protocol for reproducibility study

Three different flexible devices were submitted to an electrochemical treatment with 0.5 mol L^{-1} of sulfuric acid solution to eliminate non-conductive compounds from the printing process. Briefly, 200 µL of H_2SO_4 was added to the sensing area and cyclic voltammetry measurements were performed over the potential range from –2.5 to +2.5V (two cycles) at 100 mV s^{-1} scan rate. The sensor was rinsed with ultrapure water and the edges dried with toilet paper. Then, 6 µL of CSS suspension (1 mg mL^{-1}) was cast on the working electrode (WE) and kept at room temperature (RT) 25 °C until dry (~3h). A 6 µL aliquot of PEI (1 mg mL^{-1}) was uniformly dropped and allowed to dry at 23 ºC overnight. 20 µL of glutaraldehyde solution (1 % wt in water) was used to bond with the amine groups in PEI layer and incubated at RT 25 °C for 1 h. The devices were gently washed with ultrapure water to remove the aldehyde excess. Then, the anti-BDNF antibodies (10 µL) were immobilized in a humidity chamber at 5 °C, followed by rinsing with phosphate buffer (pH = 7.0, 0.1 M) to remove unbound antibody from the detection area. The optimized immobilization time and anti-BDNF concentration were 2 h and 2.0 µg L^{-1}, respectively. The sensors surface was blocked with 1.0 M ethanolamine solution for 1h to prevent non-specific adsorption. The BDNF immunosensor strip was stored at 4 °C until the use.

The immunosensors were incubated with 20 µL of BDNF solution (between 1.0×10^{-20} and 1.0×10^{-10} g mL^{-1}) for 30 min at room temperature, then the immunosensors were rinsed with

phosphate buffer solution to remove the unbound BDNF proteins. Finally, 150 µL of $Fe(CN)_6^{3-/4-}$ (5mM) prepared in 0.1 M of phosphate buffer solution was added to the BDNF immunosensor strip and an EIS measurement was recorded. The working frequency was between 0.1 Hz and 10000 kHz under open circuit potential with 10 mV of amplitude. Following each successive addition of BDNF, the impedimetric responses were monitored to track the changes in R_{ct} values with respect to concentration. The analytical signal was obtained from the difference between R_{ct} in presence and absence of BNDF antigen ($\Delta R_{ct} = Rct_{BDNF} - Rct_{ethanolamine}$). The semicircle in Nyquist plot correspond to the charge-transfer resistance, Rct. We used the slope from ΔR_{ct} versus $\log C_{BDNF}$ (analytical curve) of each device to plot Figure 5A, i.e., slope of each device versus number of devices.

1.4 Protocol for long-term storage stability study

Eighteen different flexible devices were submitted to an electrochemical treatment with 0.5 mol L^{-1} of sulfuric acid solution to eliminate non-conductive compounds from the printing process. Briefly, 200 µL of H_2SO_4 was added to the sensing area and cyclic voltammetry measurements were performed over the potential range from –2.5 to +2.5V (two cycles) at 100 mV s^{-1} scan rate. The sensor was rinsed with ultrapure water and the edges dried with toilet paper. Then, 6 µL of CSS suspension (1 mg mL^{-1}) was cast on the working electrode (WE) and kept at room temperature (RT) 25 °C until dry (~3h). A 6 µL aliquot of PEI (1 mg mL^{-1}) was uniformly dropped and allowed to dry at 23 ºC overnight. 20 µL of glutaraldehyde solution (1 % wt in water) was used to bond with the amine groups in PEI layer and incubated at RT 25 °C for 1 h. The devices were gently washed with ultrapure water to remove the aldehyde excess. Then, the anti-BDNF antibodies (10 µL) were immobilized in a humidity chamber at 5 °C, followed by rinsing with

phosphate buffer (pH = 7.0, 0.1 M) to remove unbound antibody from the detection area. The optimized immobilization time and anti-BDNF concentration were 2 h and 2.0 μg L^{-1}, respectively. The sensors surface was blocked with 1.0 M ethanolamine solution for 1h to prevent non-specific adsorption. The BDNF immunosensor strip was stored at 4 °C until the use.

After 7, 14, 21, 30 and 60 days of assembling the immunosensor described above, three immunosensor strips were incubated with 20 μL of BDNF solution (between 1.0×10^{-20} and 1.0×10^{-10} g mL^{-1}) for 30 min at room temperature, then the immunosensors were rinsed with phosphate buffer solution to remove the unbound BDNF proteins. Finally, 150 μL of $Fe(CN)_6^{3-/4-}$ (5 mM) prepared in 0.1 M of phosphate buffer solution was added to the BDNF immunosensor strip and an EIS measurement was recorded. The working frequency was between 0.1 Hz and 10000 kHz under open circuit potential with 10 mV of amplitude. Following each successive addition of BDNF, the impedimetric responses were monitored to track the changes in R_{ct} values with respect to concentration. The analytical signal was obtained from the difference between R_{ct} in presence and absence of BNDF antigen ($\Delta R_{ct} = Rct_{BDNF} - Rct_{ethanolamine}$). The semicircle in Nyquist plot correspond to the charge-transfer resistance, Rct. We used the slope from ΔR_{ct} versus $logC_{BDNF}$ (analytical curve) of each device to plot Figure 5B, i.e., the slope of each device versus number of days (0, 7, 14, 21, 30 and 60 days). This set of experiment was made in triplicate.

1.5 Protocol for interference study

Ten different flexible devices were submitted to an electrochemical treatment with 0.5 mol L^{-1} of sulfuric acid solution to eliminate non-conductive compounds from the printing process. Briefly, 200 μL of H_2SO_4 was added to the sensing area and cyclic voltammetry measurements

were performed over the potential range from –2.5 to +2.5V (two cycles) at 100 mV s^{-1} scan rate. The sensor was rinsed with ultrapure water and the edges dried with toilet paper. Then, 6 µL of CSS suspension (1 mg mL^{-1}) was cast on the working electrode (WE) and kept at room temperature (RT) 25 °C until dry (~3h). A 6 µL aliquot of PEI (1 mg mL^{-1}) was uniformly dropped and allowed to dry at 23 ºC overnight. 20 µL of glutaraldehyde solution (1 % wt in water) was used to bond with the amine groups in PEI layer and incubated at RT 25 °C for 1 h. The devices were gently washed with ultrapure water to remove the aldehyde excess. Then, the anti-BDNF antibodies (10 µL) were immobilized in a humidity chamber at 5 °C, followed by rinsing with phosphate buffer (pH = 7.0, 0.1 M) to remove unbound antibody from the detection area. The optimized immobilization time and anti-BDNF concentration were 2 h and 2.0 µg L^{-1}, respectively. The sensors surface was blocked with 1.0 M ethanolamine solution for 1h to prevent non-specific adsorption. The BDNF immunosensor strip was stored at 4 ℃ until the use.

One immunosensor was incubated with 20 µL of BDNF solution at 1 µg mL^{-1} for 30 min at room temperature, then the immunosensor were rinsed with 0.1 M phosphate buffer solution to remove the unbound BDNF proteins. Nine immunosensor were individually incubated with 20 µL of glucose, NaCl, paracetamol, BSA, SARS-CoV-2 S protein, urea, uric acid, ascorbic acid and lactate in phosphate buffer, i.e. one device for each interferent specie, for 30 min at room temperature, then the immunosensor were rinsed with 0.1 M phosphate buffer solution. In all ten devices, 150 µL of $Fe(CN)_6^{3-/4-}$ (5 mM) prepared in 0.1 M of phosphate buffer solution was added to the BDNF immunosensor strip and an EIS measurement was recorded. The working frequency was between 0.1 Hz and 10000 kHz under open circuit potential with 10 mV of amplitude. Following each successive addition of BDNF, the impedimetric responses were monitored to track the changes in R_{ct} values with respect to concentration. The analytical signal was obtained from

the difference between R_{ct} in presence and absence of BNDF antigen (ΔR_{ct} = Rct_{BDNF} – $Rct_{ethanolamine}$). The semicircle in Nyquist plot correspond to the charge-transfer resistance, Rct. The red bar in Figure 5C with ΔR_{ct} of 60 ohms is the reference for BDNF protein detection.

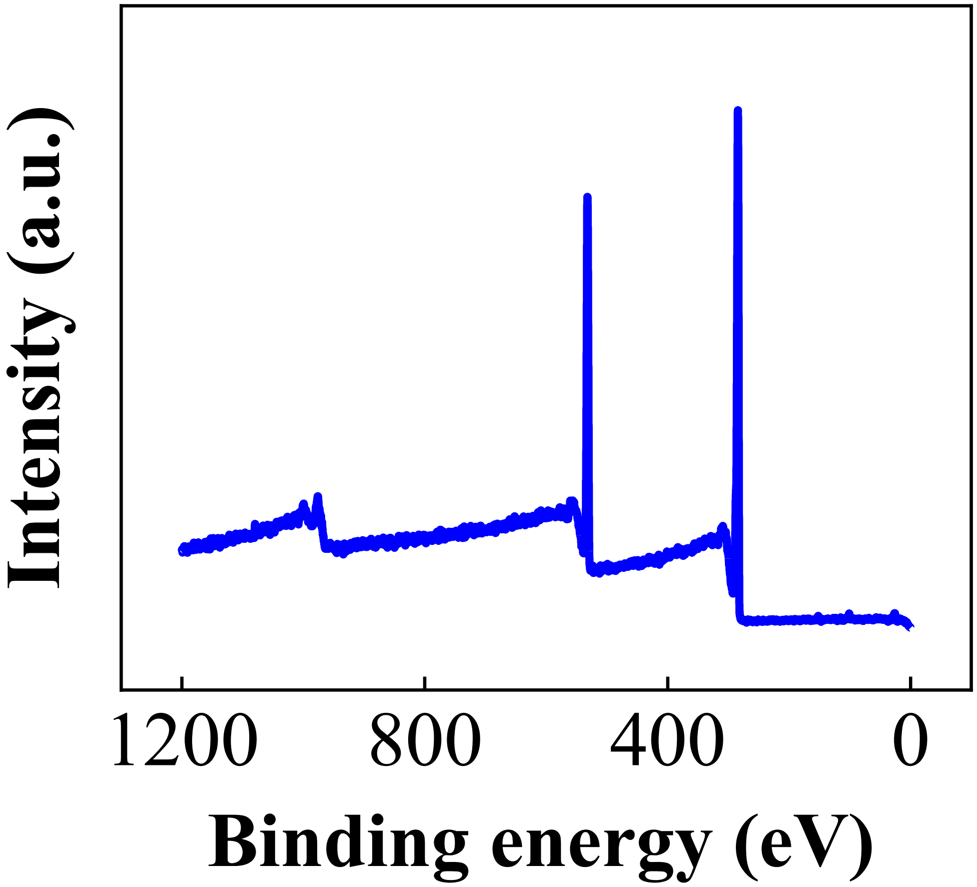

Figure S1 – XPS survey spectrum of CSS.

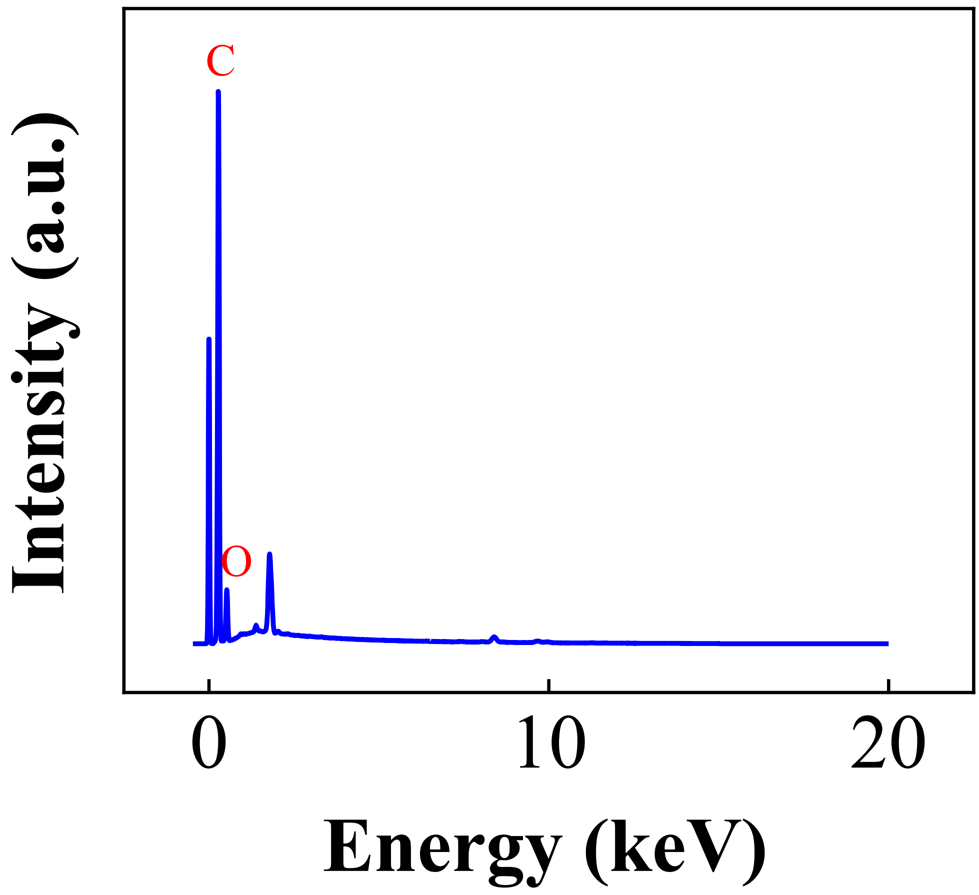

Figure S2 – EDS spectrum of CSS.
